# Caveolin-1 Influences LFA-1 Redistribution upon TCR Stimulation in CD8 T Cells

**DOI:** 10.4049/jimmunol.1700431

**Published:** 2017-06-21

**Authors:** Jessica G. Borger, Vicky L. Morrison, Andrew Filby, Celine Garcia, Liisa M. Uotila, Fabio Simbari, Susanna C. Fagerholm, Rose Zamoyska

**Affiliations:** * Institute of Immunology and Infection Research, University of Edinburgh, Edinburgh EH9 3FL, United Kingdom;; † University of Glasgow, Glasgow G12 8TA, United Kingdom;; ‡ Faculty of Medical Sciences, Newcastle University, Newcastle upon Tyne NE1 7RU, United Kingdom; and; § Institute of Biotechnology, University of Helsinki, 00014 Helsinki, Finland

## Abstract

TCR stimulation by peptide–MHC complexes on APCs requires precise reorganization of molecules into the area of cellular contact to form an immunological synapse from where T cell signaling is initiated. Caveolin (Cav)1, a widely expressed transmembrane protein, is involved in the regulation of membrane composition, cellular polarity and trafficking, and the organization of signal transduction pathways. The presence of Cav1 protein in T cells was identified only recently, and its function in this context is not well understood. We show that Cav1-knockout CD8 T cells have a reduction in membrane cholesterol and sphingomyelin, and upon TCR triggering they exhibit altered morphology and polarity, with reduced effector function compared with Cav1 wild-type CD8 T cells. In particular, redistribution of the β_2_ integrin LFA-1 to the immunological synapse is compromised in Cav1-knockout T cells, as is the ability of LFA-1 to form high-avidity interactions with ICAM-1. Our results identify a role for Cav1 in membrane organization and β_2_ integrin function in primary CD8 T cells.

## Introduction

T cells require integrin-mediated cell adhesion to interact stably with APCs and initiate optimal TCR signaling and activation (1, 2). Integrins are heterodimeric transmembrane proteins, composed of α and β subunits, which are capable of bidirectional signaling across the plasma membrane. In naive T cells, integrin binding is of low affinity, as the molecules are mainly in a low-affinity conformation. Activation through surface receptors, such as TCR by peptide–MHC (pMHC) molecules or chemokine receptor by chemokine, initiates specific intracellular signaling termed “inside-out signaling,” which drives conformational changes within the integrin subunits promoting high-affinity binding to ligand (3–5). Lateral association of integrins into clusters further promotes ligand binding avidity (6, 7). In turn, “outside-in signaling,” whereby high-affinity integrin–ligand interactions result in signal transmission into the cell to drive reorganization of the actin cytoskeleton and mediate cell spreading, increases cell–cell avidity or cell–extracellular matrix adhesion.

LFA-1 (α_L_β_2_, CD11a/CD18) and very late Ag-4 (VLA-4, α_4_β_1_, CD49d/CD29) are the major integrins expressed on T cells. LFA-1 is an important structural component of the immunological synapse (IS) formed between T cell and APCs, strengthening T cell–APC interactions and facilitating cell polarization. IS formation reduces the threshold for T cell activation during cell-mediated immune responses (8–12). Integrins play important roles not only in mediating IS formation but also in cell adhesion to the extracellular matrix, contractility, motility, and growth (13–18). Under conditions of shear flow, high-affinity LFA-1 binds ICAM-1 and -2 expressed on the endothelial cells surrounding the blood vessels, facilitating firm adhesion for T cell transmigration into lymph nodes. Therefore, active LFA-1 is critical for T cell migration into secondary lymphoid tissues and other sites of inflammation (19, 20).

Caveolin (Cav) proteins have been linked with integrin signaling in multiple cell lineages (21). There are three Cav isoforms, Cav1 and Cav2, which are coexpressed in most cell lineages, including adipocytes, endothelial cells, epithelial cells, and fibroblasts, whereas Cav3 is muscle cell specific (22, 23). Cav1 has a structural role within the plasma membrane through its direct interaction with cholesterol and lipids, maintaining lipid and cholesterol homeostasis, and is the major structural component of caveolae (24). Caveolae are specialized lipid raft microdomains regarded as dynamic signaling centers in which Cav1 facilitates a variety of cellular processes through direct protein–protein interactions with heterotrimeric G proteins, Src family tyrosine kinases, H-Ras, endothelial NO synthase, and the insulin receptor (25–27). In addition to its role in caveolae, Cav1 also functions in other subcellular locations, including the focal adhesion complex (28, 29). Initial studies failed to detect Cav1 and caveolae in lymphocytes; however, Cav1 has now been identified in B cells and T cells (30–32). Moreover, Cav1 was shown to influence naive CD8 T cell activation and cell polarity (32).

To date, there are no reports on the association of Cav1 with integrin function in T cells, and we set out to investigate whether Cav1 was involved LFA-1 function. We demonstrate that following TCR engagement, Cav1-deficient CD8 T cells had altered morphology, polarization, and reduced adhesiveness to ICAM-1 under conditions of shear flow. Additionally, there was impaired homotypic adhesion and impaired LFA-1 recruitment to the IS upon TCR/pMHC association in Cav1-deficient CD8 T cells, together with a reduction in their response to Ag. Loss of Cav1 reduced the cholesterol and sphingomyelin content of CD8 T cells, suggesting that Cav1 plays a role in membrane lipid homeostasis, which influenced the redistribution of LFA-1 and its avidity for ICAM-1. Taken together, these results identify a role for Cav1 in regulating TCR signals required for LFA-1–mediated cellular adhesion and IS formation in naive CD8 T cells.

## Materials and Methods

### Mice

Cav1-deficient (Cav1-knockout [KO]) mice have been described previously (33) and were provided by B. Nichols, Cambridge University. Cav1-KO mice were backcrossed to Rag1^−/−^ OT-1 TCR transgenic mice. CD3ε-deficient (CD3ε-KO) mice were bred in-house at the University of Edinburgh. All procedures were approved under a project license granted by the U.K. Home Office and were carried out in accordance with the institutional and ethical guidelines of the University of Edinburgh.

### Cell preparation and in vitro analysis of T cell activation

T cell suspensions were prepared from lymph nodes of OT-1 or polyclonal C57/BL6 mice. Where stated, PE-conjugated Abs were used for purification of naive CD8 T cells by negative selection. Lymph node cells were incubated with anti–CD4-PE (eBioscience) and anti–I-A/I-E-PE (BioLegend) followed by a 15-min incubation with anti-PE MACS beads (Miltenyi Biotec). Cells were added to MACS LS columns (Miltenyi Biotec) and nonbinding CD8 T cells were collected. Wild-type (WT) and Cav1-KO lymph node CD8 T cells were cultured in IMDM media (Invitrogen) supplemented with 5% FCS, l-glutamine, antibiotics, and 50 μM 2-ME. OT-1 CD8 T cells were activated by the addition of peptides SIINFEKL (N4), SIITFEKL (T4), or SIIGFEKL (G4) in the presence of CD3ε-KO spleen cells as APCs to culture media as indicated. At the end of culture, CD8 T cells were stained with the relevant Abs, run through a MACSQuant flow cytometer (Miltenyi Biotec), and analyzed with FlowJo software (Tree Star). For proliferation assays, OT-1 T cells were labeled with CellTrace Violet (CTV; Molecular Probes) for 10 min before washing and culture. Proliferation was determined by assessing CTV dilution using FlowJo software (Tree Star). For LFA-1 blocking assays, M17/4 mAb (eBioscience) was incubated in 40-μl suspensions of 100,000 cells in 96-well round-bottom plates and incubated at 37°C/5% CO_2_.

### ELISA

Cytokines were measured by ELISA with paired capture and detection Abs, anti–TNF-α Ab and anti–IL-2 Ab from eBioscience. Plates (Nunc MaxiSorp) were washed with 0.05% Tween 20 in PBS and blocked with 1% BSA in PBS. After the addition of 100 μl of 3,3′,5,5′-tetramethylbenzidine substrate solution (Sigma-Aldrich) and 100 μl of 0.1 M H_2_SO_4_, absorbance was read at 450 nm with a Laboratory Systems Multiskan Ascent plate reader.

### Flow cytometry and ImageStream analysis

For flow cytometry the following conjugated Abs were used: anti–CD69-PE, (eBioscience), anti–CD11a-PE (BioLegend), anti–TCRβ-PD/Cy7 (BioLegend), anti–CD18-PerCP (BD Biosciences), and anti–CD29-BV421 (BD Biosciences). Live/Dead Aqua and CTV dyes were from Life Technologies. Samples were acquired with a MACSQuant flow cytometer (Miltenyi Biotec) and analyzed with FlowJo software (Tree Star).

For ImageStream analysis, to accurately determine the frequency and number of 1:1 T cell/APC heteroconjugates and subsequently measure the relative level of actin recruitment to the IS, splenocytes from CD3ε-KO mice were labeled with 1 μM MitoTracker Deep Red FM (MitoTDR) and then loaded with 10^−6^ M N4 peptide for 30 min at 37°C. T cells labeled with 2.5 nM CTV and MitoTDR-labeled peptide-loaded APCs were mixed 1:1 in IMDM and at indicated time points were fixed in 2% paraformaldehyde. The cells were rinsed once, permeabilized in 0.1% Triton X-100 for 6 min at room temperature, washed three times, and incubated in BODIPY-Fl (Molecular Probes) and CD11a-PE (BioLegend) for 60 min at room temperature. Samples were then acquired on a fully ASSIST-calibrated four-laser ImageStream X MKII imaging flow cytometry system (Merck Millipore/Amins). The “high sensitivity” acquisition mode was selected and images were collected at ×60 magnification. The 488-nm (blue) laser was set to 100 mW, the 561-nm (yellow/green) laser at 100 mW, the 405-nm (violet) laser at 60 mW, and the 635-nm (red) laser was set at 120 mW to maximize signal resolution but minimize CCD camera saturation. Bright-field illumination was collected in channels 1 and 9. For the purpose of spectral compensation, single-stained samples were collected with bright-field illumination and the side scatter (758 nm) laser was turned off. Compensation was determined after acquisition using the standard wizard embedded in the IDEAS analysis software package (Merck Millipore/Amnis) and the associated single-stained raw image files (Supplemental Fig. 2F). In all cases the single fluorescent populations used for determining the coefficient of compensation were manually inspected for autofluorescent cells and regated to exclude them as appropriate. For experimental samples a minimum of 10,000 cells were collected excluding cellular debris and focal calibration beads (speed beads) using a gated threshold set on the channel 1 bright-field image. Fully stained experimental samples were then converted to compensated image files using the compensation matrix shown in Supplemental Fig. 2F. The data analysis pipeline designed to identify true 1:1 T cell/APC heteroconjugates is outlined in Supplemental Fig. 2A–C. Briefly, potential heteroconjugates were identified and gated based on being dual positive for CTV (CH7) and MitoTDR (CH11). This population was further refined using the area and aspect ratio of the bright-field channel mask (M01). T cell/APC 1:1 heteroconjugates were defined and gated as having a low aspect ratio and increased area compared with single cells. Next the total integrated intensities values for BODIPY-Fl (F-actin) and CD11a-PE were used to gate on cells that were positive for both signals prior to measuring the spatial localization within the synapse. The synapse between the T cell and APC was masked using the “interface” masking adaptation within the IDEAS analysis software. The 7-aminoactinomycin D image (CH05) was used as the cell conjugate input mask (M05) and the CTV image (CH07) as the input mask for the cell of interest. The new mask is shown in Supplemental Fig. 2D image panel “M” and is overlaid on various channel images to show its suitability to mask the synapse between two cells. The mask was generated and validated using an interactive manual process as described elsewhere (34). To measure the recruitment of BODIPY-Fl/CD11a to the IS (as defined by the interface mask), the “internalization” feature was used (35). A negative internalization value indicates that most of the signal resides outside of the synaptic area, whereas increasing positive values correlate with an increasing proportion residing within the synaptic area. Data are expressed as the median internalization score of the final gated population of BODIPY-Fl/CD11a double-positive, 1:1 T cell/APC heteroconjugates. In all cases the final cell number measured was >100 cells.

### Immunoblots

Cells were lysed in 1% Triton X-100, 0.5% *n*-dodecyl-β-d-maltoside, 50 mM Tris-HCl (pH 7.5), 150 mM NaCl, 2 mM EDTA, 1 mM NaF, and 1 mM sodium orthovanadate containing protease inhibitors. Samples were separated by NuPAGE 4–12% Bis-Tris gel electrophoresis (Life Technologies). For immunoprecipitation, lysates were immunoprecipitated with anti-Cav1 rabbit Ab (BD Biosciences) coupled to Dynabeads protein A (Life Technologies). Immunoprecipitates were washed with lysis buffer, and proteins were transferred onto polyvinylidene difluoride Immobilon-FL membranes (Millipore). Membranes were incubated in blocking reagent (LI-COR Biosciences) before Western blotting analysis with anti-Cav1 rabbit Ab (BD Biosciences) or anti-Cav2 mouse mAb (clone 65; BD Biosciences). Proteins were detected with secondary Abs goat anti-mouse A680 (Life Technologies) and goat anti-rabbit IR800 (Thermo scientific) and visualized with an infrared imaging system (Odyssey; LI-COR Biosciences). Fractionation using a subcellular fractionation kit was performed according to the manufacturer’s instructions (Thermo Scientific).

### Confocal microscopy

For adhesion of CD8 T cells to ICAM-1 for morphological studies, 10-well multispot glass slides (Hendley-Essex, Essex, U.K.) were coated overnight in 3 μg/ml ICAM-1 (R&D Systems). Wells were washed three times in PBS, and 50 μl of naive CD8 T cells in serum-free media (1 × 10^6^/ml) treated 10 min prior with 10 μg/ml anti-CD3 (clone 2C11; R&D Systems) or 5 μg/ml SDF-1α (R&D Systems) was added. Cells were left to attach for 30 min at 37°C. Wells were washed once with PBS before fixing the cells in 4% paraformaldehyde at room temperature for 60 min. The cells were rinsed once and then permeabilized in 0.1% Triton X-100 for 6 min at room temperature. Cells were washed three times in PBS/2% BSA and incubated in BODIPY-Fl (Molecular Probes), anti-Lck Ab (Cell Signaling Technology), and anti-Ezrin Ab (EC12; Abcam) for 60 min at room temperature. Cells were washed in PBS/2% BSA and further incubated with species-specific anti–F(ab′)_2_-AF488 or AF647 conjugates (Molecular Probes) for 15 min at room temperature. The secondary Ab was removed by washing three times in PBS/2% BSA and then slides were mounted with ProLong Gold anti-fade (Molecular Probes) containing 1 μg/ml DAPI to stain nuclei. Fluorescent specimens were analyzed with a Leica TCS SP5 II confocal imaging system (Leica Microsystems) with lasers exciting at 405, 488, and 647 nm with the ×63 objective using LAS AP software (Leica). All confocal analyses were multiple repeats, and at least 100 images were analyzed for each condition. Data were rendered and analyzed using Volocity 6.3 (PerkinElmer) and ImageJ (National Institutes of Health). Pearson correlation coefficient was calculated using Volocity software to determine the pairwise colocalization of the signals with a Student *t* test used to determine statistical significance between two protein data sets. Histograms of mean fluorescence intensity (MFI) signal and protein distribution along the axis of the cell were rendered using the ImageJ plug-in RGB profiler.

### RNA isolation, RT-PCR, and quantitative real-time PCR

Tissue was isolated from the indicated organs of one mouse, added to TRIzol (Invitrogen), and homogenized in a TissueLyser II (Qiagen). RNA was extracted using 1:5 chloroform. Isoproanol was added ∼1:1 to the aqueous phase with 30 μg GlycoBlue (Invitrogen). Following a 10-min incubation at room temperature, the centrifuged pellet was washed in 75% EtOH and allowed to dry. Pellets were resuspended in 20 μl of DNase/RNase-free water and quantitated on the RNA-40 program of the NanoDrop (NanoDrop Instruments). Reverse transcription of RNA was performed with miScript II (Qiagen). Quantitative PCR was performed with SYBR Green (Qiagen) using the following primer pairs, Cav1 forward, 5′-AACGACGACGTGGTCAAGA-3′, reverse, 5′-CACAGTGAAGGTGGTGAAGC-3′; Cav2 forward, 5′-ATGACGCCTACAGCCACCACAG-3′, reverse, 5′-GCAAACAGGATACCCGCAATG-3′; polymerase chain 1 and transcript release factor (PTRF) forward, 5′-GAAAGAAAGGCGAGTGA-3′, PTRF reverse, 5′-TAATGTGTAAGTGCC CCTG-3′, and normalized to the abundance of 18S mRNA.

### Aggregation assay

CD8 T cells were resuspended in IMDM supplemented with 10% FCS and 40 mM HEPES at a cell density of 15 × 10^6^ cells/ml and aliquoted into 96-well plates at 200 μl/well. Cells were activated in 50 ng/ml phorbol dibutyrate (PdBu; Sigma-Aldrich) or 1 μg/ml anti-CD3 mAb (clone 2C11; R&D Systems) for 60 min to allow for conjugate formation. Samples (20 μl) were taken in triplicate and the number of free cells counted. Aggregation percentage was calculated as previously described in Fagerholm et al. (36); that is, the number of free cells after stimulation subtracted from the number of free cells in unstimulated samples, divided by the number of free cells in the unstimulated samples.

### Cell attachment assays

For static adhesion assays, ICAM-1 (6 μg/ml; R&D Systems) was coated onto 96-well MaxiSorp plates (Nunc) by overnight incubation at 4°C, and the wells were blocked with 1% milk in PBS. Lymphocytes from Cav1-WT or Cav1-KO mice were resuspended in adhesion medium (RPMI 1640 supplemented with 0.1% BSA, 40 mM HEPES, and 2 mM MgCl_2_) and added to the plate. Where appropriate, cells were stimulated with 200 nM PdBu (Sigma-Aldrich), 10 μg/ml anti-CD3 (R&D Systems), or 1 μg/ml SDF-1α (R&D Systems) immediately before being added to the plate. Cells were allowed to adhere for 30 min at 37°C. Unbound cells were removed by gentle washing in PBS plus 2 mM MgCl_2_, and bound cells were lysed and detected with phosphatase substrate (Sigma-Aldrich).

For shear flow adhesion assays, VI^0.4^ μ-slides (Ibidi) were coated with 6 μg/ml murine ICAM-1 (R&D Systems) overnight at 4°C. Where indicated, SDF-1α (R&D Systems) was coimmobilized by addition of 5 μg/ml onto plates for a further 30 min at 37°C. T cells were prepared at a density of 1 × 10^6^ cells/ml in binding medium (RPMI plus 0.1% BSA, 40 mM HEPES, and 2 mM MgCl_2_) and, where indicated, were stimulated with 10 μg/ml anti-CD3 (clone 2C11; R&D Systems) for 5 min. Cells were injected into a flow system that used a silicone tubing loop connected to a Multi-Phaser NE-1000 syringe pump (New Era Pump Systems), allowing the cells to flow at a continuous shear flow rate of 0.3 dyne/cm^2^. Cells were monitored by microscopy during a 10-min period, and the number of adhered cells in the field of view was determined at 1-min intervals by manual counting.

### LFA-1 affinity modulation assay

CD8 T cells (4 × 10^6^/ml) were washed and resuspended in HEPES buffer (20 mM HEPES, 140 mM NaCl, 2 g/l glucose) with 0.1% BSA. Where required, 96-well flat-bottom flexiwell plates were coated with 10 μg/ml anti-CD3 (clone 2C11; R&D Systems) at 4°C overnight. T cell aliquots of 50 μl (2 × 10^5^) were added to flexiwell plates (Dynatech, West Sussex, U.K.) with or without 5 mM Mg^2+^ and 1 mM EGTA. Soluble murine ICAM-1–Fc (10 μg/ml) was added to the cells in addition to 50 ng/ml PdBu, 100 μM N4, or 2 μg/ml anti-CD28 (R&D Systems). After 30 min incubation at 37°C, T cells were washed twice in ice-cold assay buffer with 0.1% BSA and incubated with 0.1 μg/ml Fc-specific FITC-conjugated goat anti-human IgG (Jackson ImmunoResearch Laboratories, West Grove, PA) for 20 min on ice. Cells were washed twice in ice-cold assay buffer with 0.1% BSA to remove excess unbound mAb and fluorescence was detected with a MACSQuant (Miltenyi Biotec) flow cytometer.

### Liquid chromatography–mass spectrometry analysis

Sphingolipids were first extracted from 60 μg of purified CD8 T cells (total protein amount). After injection, samples were recovered and sphingolipids were extracted after alkaline saponification (1 M KOH in MeOH). Prior to the lipid extraction, 0.2 nmol synthetic standards (Avanti Polar Lipids) were used to spike each sample to normalize the results and to obtain a relative quantification. As internal nonnatural sphingolipid standards, *N*-dodecanoylsphingosine and *N*-dodecanoylglucosylsphingosine were used for ceramide and glucosyl-ceramide, respectively, and *N*-dodecanoylsphingosyl phosphorylcholine was the standard chosen for sphingomyelin.

### Cholesterol assay

Cholesterol levels were measured in purified CD8 T cells (5 μg protein content) using the Amplex Red cholesterol assay kit (Life Technologies) according to the manufacturer’s instructions.

### Statistical analysis

A Student *t* test (paired or unpaired, two tails) and ANOVA were performed using Prism 5.0 (GraphPad Software).

## Results

### Cav1 regulates the lipid composition and Ag response of CD8 T cells

**FIGURE 1.**
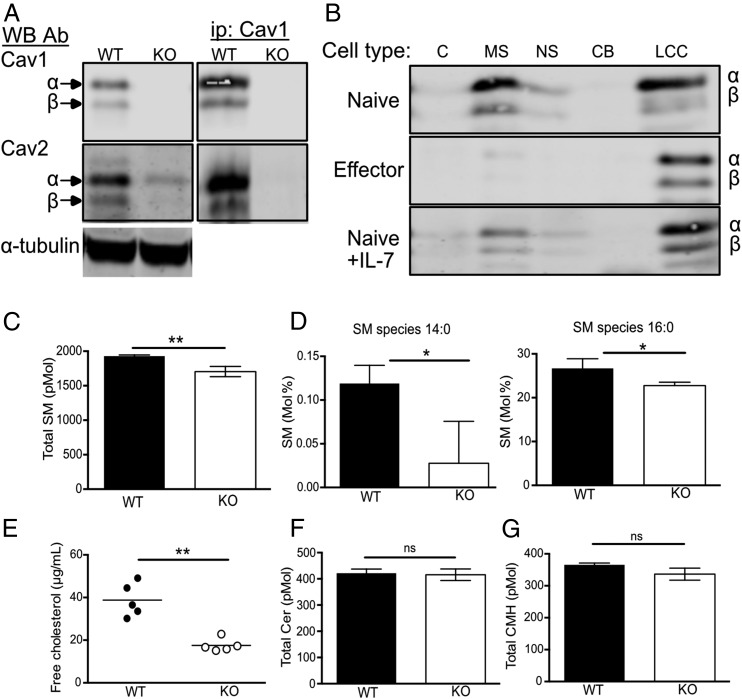
Cav1 is localized within soluble and insoluble membrane fractions and regulates cholesterol and lipid composition. (**A**) Cav1 and Cav2 protein expression was compared between WT and Cav1-KO CD8 T cell lysates by Western blot with anti-Cav1 and Cav2 Ab. Cell lysates were immunoprecipitated with anti-Cav1 and Western blots were probed sequentially with anti-Cav1 and Cav2 Ab. (**B**) Cell lysates were separated into subcellular fractions (C, cytoplasmic; MS, membrane soluble; NS, nuclear soluble; CB, chromatin bound; LCC, lipid-cytoskeletal complexes) and analyzed by Western blot with anti-Cav1 Ab. (**C**) Total sphingomyelin content in WT versus Cav1-KO CD8 T cells. (**D**) Sphingomyelin species 14:0 and 16:0 are represented as a percentage of total sphingomyelin. (**E**) Free cholesterol from WT and Cav1-KO CD8 T cells is representative of one of three independent experiments from a total of 12 biological samples of each genotype. (**F**) Total ceramide and (**G**) ceramide monohexamide content. Lipidomics data are pooled from three biological repeats. Data are shown as mean + SD. *p ≤ 0.05, **p ≤ 0.01 (Student *t* test). ns, not significant.

Cav1 was recently shown to be expressed in T cells, having previously been described as absent in T cell lines (37–40), and was shown to influence actin polymerization, synaptic membrane raft polarity, and CD8 T cell function (32). It was unclear whether T cells also expressed Cav2, which is frequently hetero-oligomerized with Cav1. Cav1 is a homodimeric transmembrane protein expressed in two isoforms, Cav1α and Cav1β, derived from alternate translation–initiation sites commencing transcription from a methionine at positions 1 and 32, respectively (41). Comparing lysates of purified CD8 T cells from naive C57BL/6 WT and Cav1-KO CD8 T cells, we confirmed that both isoforms of Cav1 were present in WT cells with a preponderance of the α isoform (Fig. 1A). Both isoforms, α and β, of Cav2 were also expressed in naive WT CD8 T cells. Cav2 could be immunoprecipated with Cav1, as described in other cell types (22). The abundance of Cav2 was greatly reduced in Cav1-KO CD8 T cells (Fig. 1A), consistent with reports showing that when not oligomerized with Cav1, Cav2 was rapidly degraded by the proteosome (42). Cav1, Cav2, and the other critical component of caveolae, cavin/PTRF, were expressed in considerably lower abundance in the thymus and secondary lymphoid organs compared with lung and adipose tissue (Supplemental Fig. 1A–C). As previously suggested (37–40), we could not identify any caveolae-like structures in naive CD8 T cells whereas they could be seen by electron microscopy in A431 cells (Supplemental Fig. 1D), confirming that Cav1 in T cells was not associated with caveolae.

Cav1 is found within all membranes of the cell and is also involved in the shuttling of cholesterol from the endoplasmic reticulum to the plasma membrane, directing Cav1 to cholesterol and sphingolipid-enriched membrane domains (43, 44). We asked where Cav1 localized within naive CD8 T cells and found that ∼50% of Cav1 protein localized within the membrane-soluble fraction of naive CD8 T cells, with the remaining Cav1 protein found within the insoluble cell pellet, containing lipid–cytoskeletal complexes (Fig. 1B). Upon in vitro stimulation of T cells with anti-CD3 Ab for 48 h, Cav1 wholly redistributed within lipid–cytoskeletal complex fractions (Fig. 1B). Redistribution of Cav1 appeared to be driven by in vitro TCR activation, as it was less evident in cells cultured in the cytokine IL-7, which maintains T cell viability without stimulating proliferation (Fig. 1B).

Given that Cav1 directly binds cholesterol and specific sphingolipids, we asked whether the lipid composition was altered in naive CD8 T cells in the absence of Cav1 by performing lipidomic analysis. Sphingomyelin, one of the most abundant sphingolipids in cells and a major component of lipid rafts, was significantly reduced in naive CD8 T cells in the absence of Cav1 (Fig. 1C). Furthermore, analysis of individual sphingomyelin species revealed a marked reduction in short fatty acids in Cav1-KO CD8 T cells (Fig. 1D). Additionally, there was a significant decrease in the abundance of free cholesterol in Cav1-KO naive CD8 T cells compared with WT cells (Fig. 1E). In contrast, Cer and GluCer, other classes of sphingolipids, were not reduced in the absence of Cav1 (Fig. 1F, 1G), which suggested a role for Cav1 in the transport of specific lipids to the plasma membrane in CD8 T cells.

**FIGURE 2.**
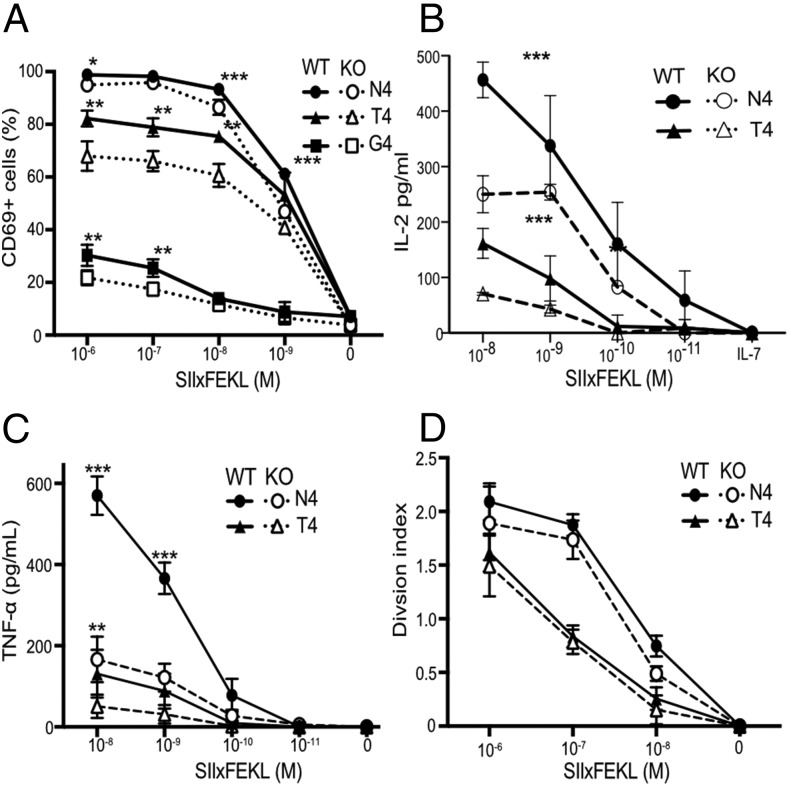
Cav1 regulates CD8 T cell responses to Ag. (**A**) The frequency of CD69^+^ cells among Cav1-WT and Cav1-KO OT-1 CD8 T cells stimulated for 3 h with a titration of N4, T4, or G4 peptides. (**B** and **C**) Cav1-WT and Cav1-KO OT-1 CD8 T cells were stimulated and supernatants were assayed at 24 h for total IL-2 (B) and TNF-α (C) by ELISA. (**D**) Proliferation at 72 h of CTV-labeled OT-1 T cells is represented by the division index calculated using FlowJo software. Data are representative of two independent experiments, each performed on three mice per group. Error bars represent SD. *p ≤ 0.05, **p ≤ 0.01, ***p ≤ 0.001 (Student *t* test, corrected for multiple comparisons using the Holm–Sidak method).

The abundance of cholesterol in the plasma membrane has been linked directly to the ability of CD8 T cells to respond to Ag (45), so we crossed Cav1-KO mice onto the OT-1 class 1 MHC–restricted, TCR transgenic background to enable interrogation of Ag-specific CD8 T cell responses. Responses of Cav1-WT and Cav1-KO OT-1 CD8 T cells were measured to a range of peptides of differing affinities: N4, a strong agonist; T4, a partial agonist; and G4, a very weak agonist. We confirmed that loss of Cav1 impaired the response of CD8 T cells to TCR/pMHC stimulation in vitro (Fig. 2), as previously reported for polyclonal CD8 T cells stimulated with anti-CD3/28 Abs (32). The absence of Cav1 had a significant impact on the upregulation of the early activation marker CD69 for all altered peptide ligands (Fig. 2A) and resulted in a significant reduction in the development of effector function, as measured by the production of cytokines IL-2 and TNF-α (Fig. 2B, 2C) and, as previously described (32), IFN-γ (data not shown). Although activation and effector functions were significantly impacted by Cav1 deficiency, there was only a subtle reduction in proliferation in Cav1-KO CD8 T cells (Fig. 2D). At first sight, these data appear to contradict a previous report (32) showing that Cav-KO CD8 T cells have reduced proliferation to anti–CD3 plus CD28 stimulation in vitro and expansion to lymphocytic choriomeningitis virus in vivo. However, Tomassian et al. (32) showed that whereas a reduced proportion of Cav-KO CD8 T cells had entered division by 48 h, there was no defect in their ability to undergo multiple divisions. In contrast to anti–CD3 plus CD28 stimulation, the strong agonist N4 peptide drives all the OT-1 T cells synchronously into division in vitro, and therefore the strength of the stimulus would be likely to minimize any differences in proliferation between WT and KO T cells so that although there was a trend for less proliferation in the Cav1-KOs, this was not significant over multiple experiments. Overall, our data confirm that even though Cav1 was expressed at much lower abundance in T cells than in nonlymphoid tissues and was not associated with caveolae, Cav1 influenced the lipid composition of CD8 T cell membranes, and its absence impaired the activation and the development of effector function of CD8 T cells upon stimulation with Ag.

### Cav1 influences CD11a recruitment to the IS

**FIGURE 3.**
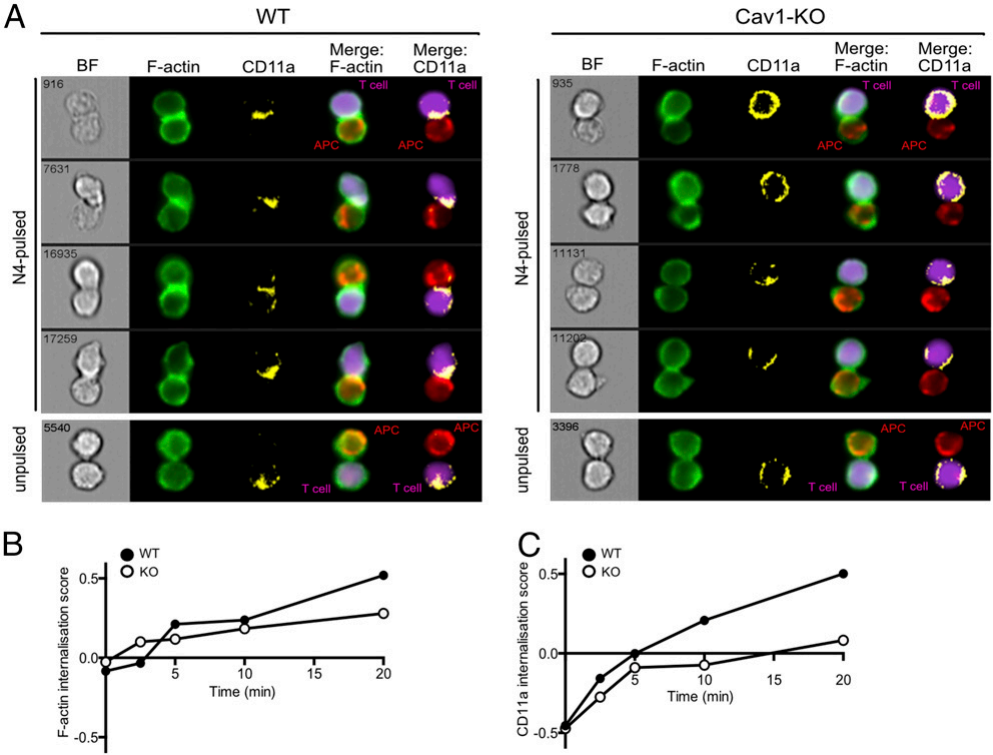
Impaired recruitment of F-actin and CD11a to the IS in the absence of Cav1. ImageStream analysis assessing conjugate formation of Cav1-WT or Cav1-KO OT-1 CD8 T cells with N4-pulsed APCs for the indicated times (0–20 min) is shown. Original magnification ×60. Conjugates were fixed, permeabilized, and stained for BODIPY-Fl (F-actin, green) and CD11a (yellow). (**A**) The four rows show representative cells at 20 min, with distribution of BODIPY-Fl and CD11a within the synapse formed between T cells (purple) and APCs (red) shown in merge columns 4 and 5, respectively. Graphs of the internalization method, previously described in (35), for Cav1-WT and Cav1-KO conjugates stained with (**B**) BODIPY-Fl and (**C**) CD11a are representative of three independent experiments.

A critical first step in the activation of T cells is the formation of an IS, a specialized junction between the T cell and the APC. Recent evidence has shown that the IS comprises a highly dynamic periphery in which TCRs in microvesicles engage with intracellular signaling molecules (8) whereas the structure is stabilized by a peripheral ring (pSMAC) composed of the integrin LFA-1 interacting with its ligand ICAM1. To assess whether the reduced activation of Cav1-KO CD8 T cells was linked to changes in IS formation, we used a high-throughput imaging flow cytometer, ImageStream, to obtain a measure of the spatial distribution of LFA-1 (CD11a) from a large number of conjugates over multiple early time points. Details of spillover coefficients and the analytical and masking workflow used in the analysis are provided in Supplemental Fig. 2. Both Cav1-WT and Cav1-KO naive OT-I CD8 T cells formed conjugates with N4-pulsed APCs during the 20-min observation period compared with unpulsed controls (Fig. 3A). LFA-1 surface expression was homogeneously dispersed on unconjugated CD8 T cells and on conjugates formed with APCs in the absence of Ag (unpulsed) in both control and Cav1-KO CD8 T cells. Upon T cell conjugation with N4-pulsed APCs, both F-actin and CD11a redistributed to the IS in Cav1-WT and Cav1-KO CD8 T cells. Calculation of the internalization score, the amount of protein within the cell–cell contact area, revealed a reduction of both F-actin and CD11a recruitment to the IS in the absence of Cav1 (Fig. 3B, 3C), as seen clearly at the 20 min time point shown in Fig. 3A.

### Cav1 alters T cell morphology and polarization

**FIGURE 4.**
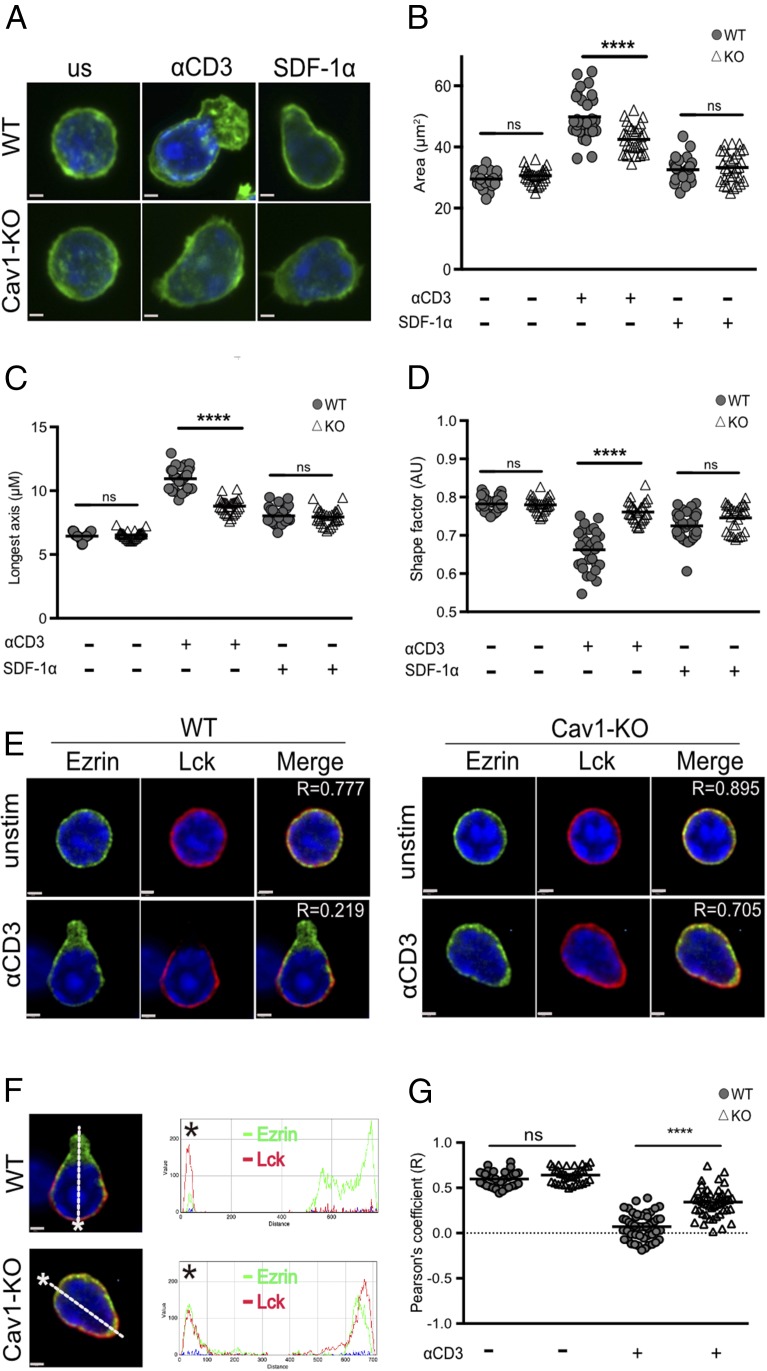
Absence of Cav1 alters CD8 T cell morphology and polarization upon adhesion to ICAM-1. Cav1-WT and Cav1-KO OT-1 CD8 T cells were allowed to adhere for 30 min to glass slides with and without 3 μg/ml ICAM-1 coating. (**A**) Cells were stained with BODIPY-Fl (green) to define the cell perimeter. Cav1-WT and Cav1-KO CD8 T cells spreading on ICAM-1 were quantified in terms of (**B**) area, (**C**) longest axis, and (**D**) shape factor. For shape factor, a value of 1 is circular. (B–D) Small horizontal lines indicate the mean. (**E** and **F**) Cells were stained with Ezrin (green) and Lck (red). Merge images were sectioned (white line) from the leading edge, indicated by the asterisk, to the uropod, generating an RGB histogram using ImageJ software. The histogram line indicates the distribution and MFI of proteins throughout the cross-section of the cell. (**G**) A Pearson correlation coefficient was calculated by Volocity software, and *p* values were calculated by the Student *t* test. Data are representative of one of three independent experiments with a minimum of 100 cells per condition. Scale bars, 1 μm. ****p ≤ 0.001.

Less LFA-1 was recruited to the IS in the absence of Cav1, which could result from Cav1 directly influencing LFA-1 signaling, as Cav1 is known to influence integrin signaling in other cell types (46–48), or as an indirect effect arising from defective TCR stimulation of inside-out signaling to LFA-1. To better understand how lack of Cav1 influenced LFA-1 interactions with ICAM-1, we looked at the consequences on cell morphology and distribution of particular intracellular signaling molecules following LFA-1 interaction with immobilized ICAM-1. Naive OT-1 CD8 T cells require either TCR or chemokine CXCR4 stimulation to induce a conformational change in LFA-1 to its active, high-affinity conformation. Cav1-WT and Cav1-KO CD8 T cells were activated with anti-CD3 Ab or SDF-1α (CXCL12), the ligand for CXCR4, for 10 min prior to addition to glass slides coated with ICAM-1. Resting T cells have a spherical morphology with F-actin homogeneously distributed around the plasma membrane, as shown by both Cav1-WT and Cav1-KO CD8 T cells (Fig. 4A). After 10 min of anti-CD3 stimulation, Cav1-WT and Cav1-KO CD8 T cells were attached to ICAM-1–coated glass coverslips for 30 min, whereupon Cav1-WT cells became polarized and formed the characteristic F-actin extensions at one pole. However, Cav1-KO CD8 T cells lacked a defined uropod structure (Fig. 4A). Calculation of the surface area of the anti–CD3 Ab-stimulated cells showed that Cav1-KO CD8 T cells had spread significantly less, with a reduced area and mean of the longest axis, than did their WT counterparts (Fig. 4B, 4C). Accordingly, the shape factor was significantly lower in Cav1-KO CD8 T cells, as the cells were more circular than the WT cells, which formed distinct uropod protrusions (Fig. 4D).

Cav1 deficiency has been reported to cause Src-dependent removal of focal adhesions from cell edges in fibroblast cells (49). We therefore asked whether loss of morphology was indicative of aberrant polarity of TCR-stimulated CD8 T cells upon LFA-1 interaction with ICAM-1. Ezrin, one of the ezrin-radixin-moesin proteins that links the plasma membrane to the actomyosin cortex and modulates T cell polarization through regulating cortical tension (50), was found to be concentrated within the defined uropod of Cav1-WT CD8 T cells following TCR stimulation (Fig. 4E). Strikingly, Ezrin remained homogeneously localized around the cell periphery in Cav1-KO CD8 T cells. Loss of Lck clustering with the TCR following early activation of Cav1-KO regulatory T cells was reported recently (51), suggesting that Lck localization at the leading edge of migrating T cells could be impeded in Cav1-KO CD8 T cells. Indeed, we found that although Cav1-WT CD8 T cells polarized Lck to the leading edge, in Cav1-KO cells Lck maintained a homogenous distribution around the cell membrane. To visualize the proportion of molecules that redistributed toward the uropod and leading edge of TCR-stimulated CD8 T cells, we used spectral overlaps and RGB histogram analysis. A merge image sectioned from the leading edge (designated by an asterisk) to the opposing uropod (trajectory represented by a white line) showed that in Cav1-WT CD8 T cells, most Lck was polarized to the leading edge of the migrating cell, with most Ezrin molecules concentrated within the uropod (Fig. 4F). Analysis of Cav1-KO CD8 T cells identified a similar proportion of Lck and Ezrin molecules within both the leading edge and uropod. A comparison of the Pearson correlation coefficient between Lck and Ezrin revealed that following TCR stimulation, Cav1-WT CD8 T cells have a significantly lower level of protein colocalization than do Cav1-KO cells (Fig. 4G). Taken together, these results show that Cav1 plays a role downstream of the TCR in maintaining T cell morphology and directing cellular polarization upon LFA-1–mediated cellular adhesion of CD8 T cells.

### Impaired adhesion to ICAM-1 under conditions of shear flow in the absence of Cav1

**FIGURE 5.**
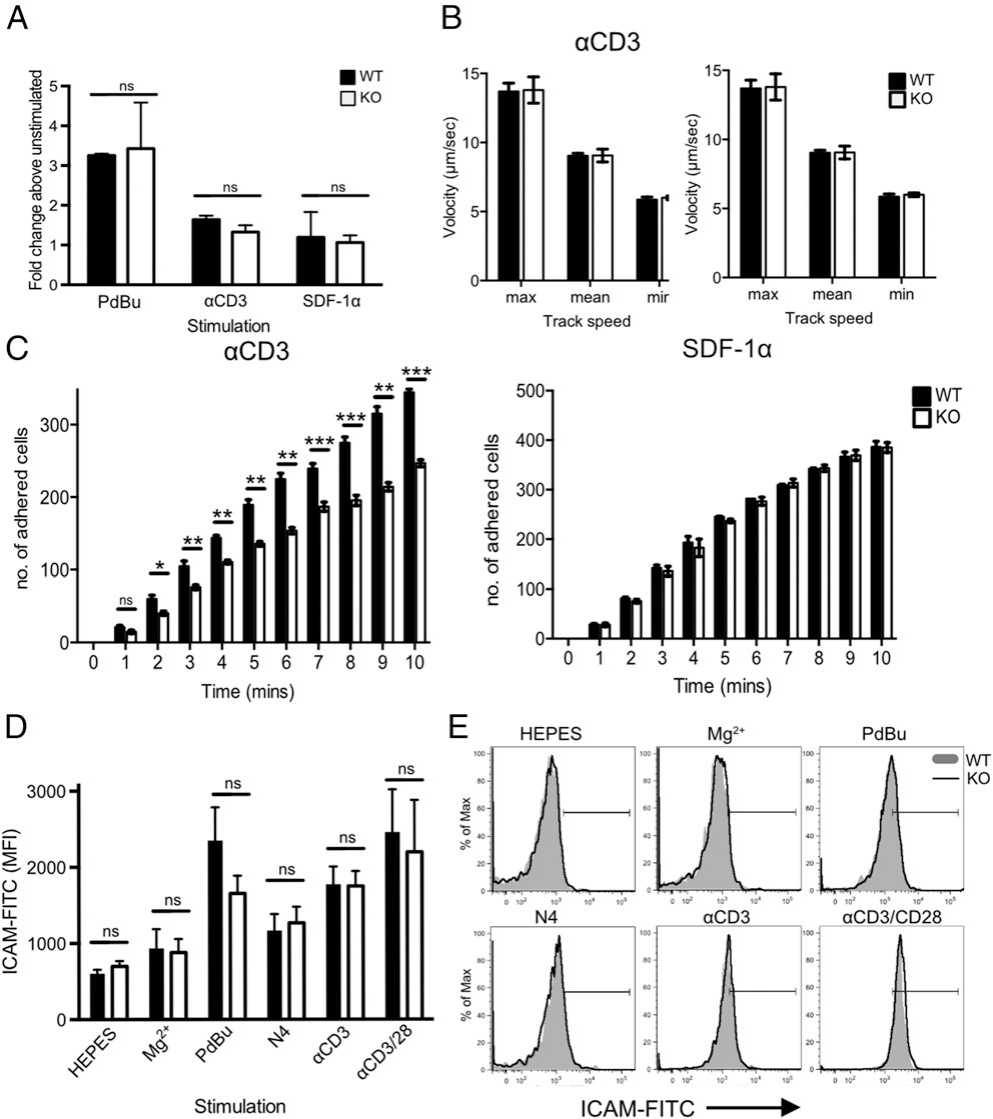
Impaired adhesion to ICAM-1 under conditions of fluid shear stress in the absence of Cav1. (**A**) Static adhesion of WT (filled bars) versus Cav1-KO (open bars) CD8 T cells to ICAM-1. CD8 T cells were stimulated with 50 nM PdBu, 1 μg/ml anti-CD3 Ab, or 5 μg/ml SDF-1α before the start of the assay. Fluid shear flow rates were set at 0.3 dyne/cm^2^. (**B**) Rolling rates of the cells were analyzed in parallel with (**C**) the number of adherent cells. Data are shown as mean ± SEM of data pooled from two independent experiments, each performed in triplicate on two mice per group. *p ≤ 0.05, **p ≤ 0.01, ***p ≤ 0.001 (Student *t* test). (**D**) Cav1-WT or Cav1-KO OT-1 CD8 T cells were incubated for 30 min with chimeric ICAM-1 in HEPES buffer alone or supplemented with 100 nM Mg^2+^, or stimulated with PdBu, N4 peptide, anti-CD3, or anti-CD3 plus anti-CD28, as indicated. ICAM-1 bound to T cells was detected by staining with human IgG-FITC. ICAM-1 MFI ± SEM from one of three independent experiments. (**E**) Representative histograms from each condition, as indicated. ns, not significant.

The altered morphology and polarization of Cav1-KO CD8 T cells interacting with ICAM-1 led us to first analyze cell adhesion using a static adhesion assay in which the capacity of cells to adhere to ligand in the absence of shear flow is quantified. Adhesion of Cav1-KO CD8 T cells to immobilized ICAM-1 was comparable to that observed in WT cells, whether cells were untreated or stimulated with phorbol esters (PdBu), TCR, or chemokine ligands (Fig. 5A).

Integrins mediate cell adhesion under shear flow conditions and are essential for the adhesion of T cells to high endothelial venules, a process that is dependent on high-affinity LFA-1 interactions with ICAM-1. Both external (shear-based) and internal (cytoskeleton-based) forces contribute to LFA-1–ICAM-1 interactions and integrin activity (52). We asked whether the absence of Cav1 influenced adhesion of naive CD8 T cells to immobilized ICAM-1 under conditions of fluid shear stress. Following preincubation with anti-CD3 or SDF-1α, T cells were allowed to flow over plates coated with ICAM-1 alone at the shear rate of 0.3 dyne/cm^2^. The initial transient low-affinity interaction between T cells and endothelium in vivo is mediated mainly through P- and L-selectin and results in T cell rolling. We did not include selectins in this assay so that we could assess rolling as a direct measure of low to intermediate affinity LFA-1–ICAM-1 interactions, which occur at strengths too weak to allow firm attachment. Rolling rates of WT and Cav1-KO CD8 T cells were comparable following either TCR or SDF-1α stimulation (Fig. 5B), indicating that these weak LFA-1–ICAM-1 affinities were unaffected by the loss of Cav1. However, when we tested whether the cells could form firm attachments by measuring the number of adherent cells under conditions of flow, we found that the Cav-1KO CD8 T cells were 40% less adherent than WT cells to ICAM-1 (Fig. 5C). This difference was only observed following anti-CD3 stimulation of the cells and was not apparent following stimulation with the chemokine SDF-1α. These data indicate that the TCR signals that license high-avidity interactions of LFA-1 with ICAM-1 are compromised in Cav1-KO CD8 T cells.

High-avidity interactions between LFA-1 and ICAM-1 are the result of both conformational changes in the LFA molecules themselves, which lead to higher affinity binding termed “affinity modulation,” and changes in local integrin density, which increase valency and result in “avidity modulation.” To ask whether loss of Cav1 impaired LFA-1 affinity modulation, we first examined naive OT-1 CD8 T cell adhesion to soluble ICAM-1 following activation of LFA-1 directly. Treatment of the cells with the divalent cation Mg^2+^ in the presence of EGTA, to prevent Ca^2+^-dependent changes in avidity, can be used to increase LFA-1 affinity (53, 54). When LFA-1 was in a low-affinity state (HEPES buffer only) the amount of soluble ICAM-1 binding was low and equivalent in Cav1-WT and Cav1-KO CD8 T cells (Fig. 5D, 5E). Addition of Mg^2+^/EGTA caused only a negligible and comparable MFI shift in ICAM-1 binding to both Cav1-WT and Cav1-KO CD8 T cells. We next addressed the ability of CD8 T cells to bind ICAM-1 following stimulation with PdBu, peptide (N4), anti-CD3 Ab or anti-CD3 plus soluble anti-CD28 Abs (CD3/28). After 30 min stimulation, adhesion of soluble ICAM-1 was enhanced in a comparable manner for Cav1-WT and Cav1-KO CD8 T cells and both bound ICAM-1 maximally following stimulation with PdBu and anti-CD3/28 treatment. These data indicate that there is no defect in upregulation of LFA-1 affinity following TCR stimulation in Cav1-KO CD8 T cells and suggest instead that compared with Cav1-WT, Cav1-KO cells may not be able to redistribute LFA-1 as effectively to promote high-avidity LFA-1–ICAM-1 interactions.

### Impaired LFA-1–mediated aggregation in the absence of Cav1

**FIGURE 6.**
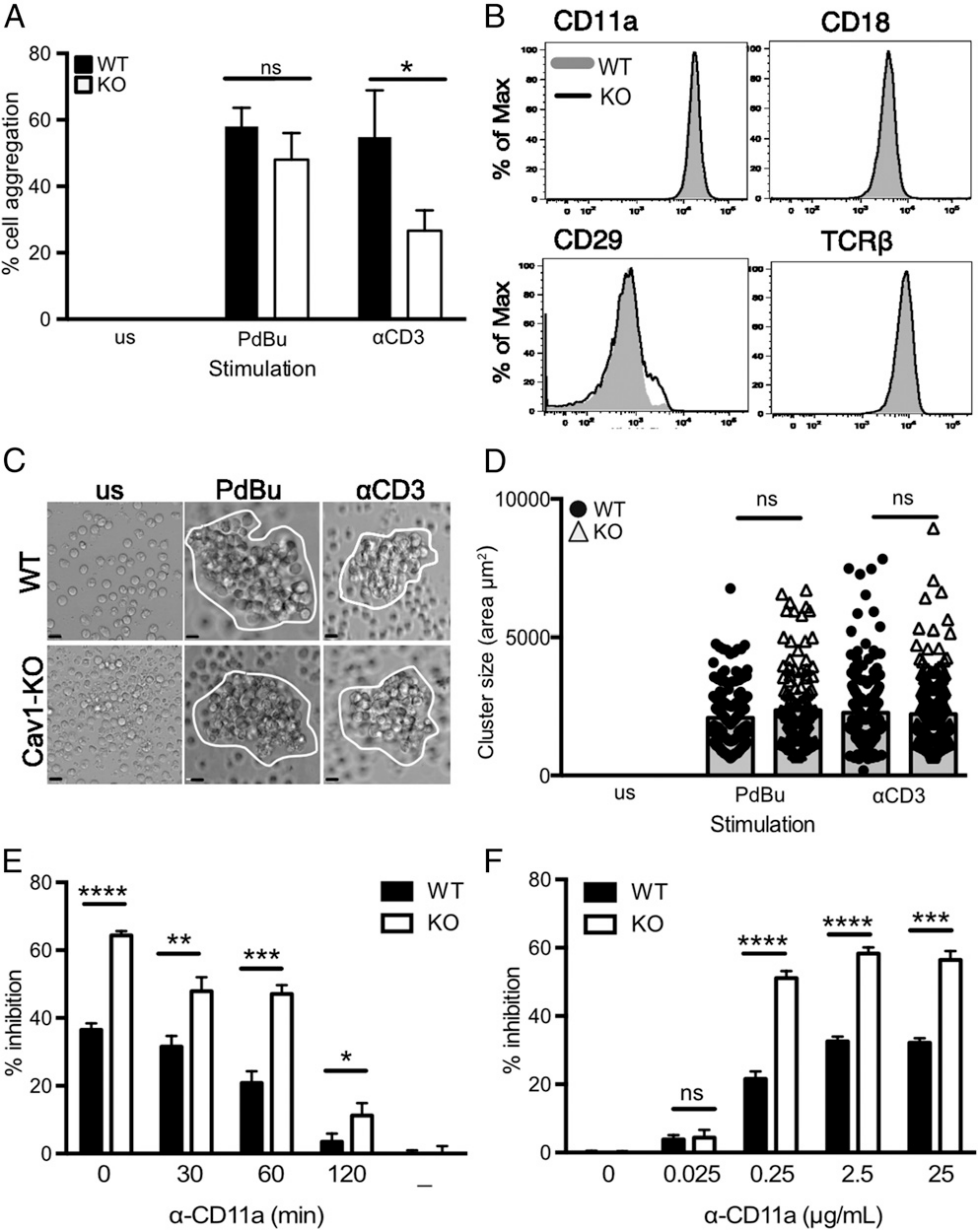
Impaired LFA-1–mediated CD8 T cell homotypic aggregation in the absence of Cav1. (**A**) Purified Cav1-WT (filled bars) and Cav1-KO (open bars) OT-1 CD8 T cells were activated with 50 ng/ml PdBu or 1 μg/ml anti-CD3 for 60 min and assessed for cell–cell aggregation. Data are shown as a mean ± SEM of data representing one of five independent experiments with each count performed in triplicate on two to four mice per group. (**B**) Expression levels of the indicated surface proteins in WT (gray shaded) and Cav1-KO (black line) naive OT-1 CD8 T cells. Data are representative of 25 mice per group. Naive CD8 T cells were cultured for 24 h and then (**C**) imaged and (**D**) assessed for cluster size using Volocity software. Data are pooled from two independent experiments with a minimum of 100 cells per condition (mean and SD). Scale bars, 5 μm. (**E**) Anti–LFA-1 blocking mAb was added at various time points or (**F**) at various concentrations, following 1 μM N4 peptide stimulation of Cav1-WT and Cav1-KO CD8 T cells. Inhibition of CD69 upregulation (percentage inhibition) was measured at 3 h. Data are representative of one of three independent experiments. *p ≤ 0.05, **p ≤ 0.01, ***p ≤ 0.001, ****p = 0.0001 (Student *t* test); ns, not significant.

ICAM-1 is expressed on the surface of T cells, and in culture LFA-1 mediates cell–cell adhesion by binding in *trans* to this ligand in response to stimuli, including PdBu (55, 56). Thus, activation of LFA-1 can lead to the formation of aggregates of homotypically adherent cells (57), which is considered to be a reflection of ligand-dependent redistribution of LFA-1 molecules (58). We examined the ability of purified CD8 T cells to form aggregates following stimulation with anti-CD3 or with PdBu plus ionomycin, which activates integrins while bypassing TCR-proximal signaling (Fig. 6A). The percentage of naive OT-1 CD8 T cells that formed aggregates after 60 min was significantly lower in Cav1-KO T cell cultures compared with Cav1-WT cultures in response to anti-CD3. In contrast, PdBu plus ionomycin stimulation caused equivalent aggregation. Reduced aggregation in the absence of Cav1 was not due to altered expression of adhesion molecules on Cav1KO CD8 T cells, as phenotyping for surface markers confirmed that there was equivalent abundance of both the α_L_ (CD11a) and β_2_ (CD18) chains of LFA-1, as well as other adhesion molecules, including the β_1_ integrin CD29 (VLA4) together with the TCR complex (Fig. 6B). By 24 h after activation, CD8 T cells formed large clusters that were comparable in size between Cav1-KO and Cav1-WT cells (Fig. 6C, 6D). These data indicate that in CD8 T cells, Cav1 plays a role in LFA-1–ICAM-1-dependent cell interactions early after stimulation but it does not impact later aggregate formation, consistent with Cav1 impairing some aspects of T cell activation (Fig. 2).

LFA-1–mediated homotypic aggregation has been linked to optimal activation of T cells in vitro (59–61), and combined with our observations that there was reduced LFA-1 recruitment to the IS (Fig. 3) suggested that a failure to form early stable LFA-1–ICAM-1 interactions could contribute to the impaired responses to Ag of Cav1-KO OT-1 CD8 T cells. To assess this, we examined the effect on activation with N4 peptide of disrupted T cell/APC conjugates by addition of blocking LFA-1 mAb. CD69 upregulation, normalized against cells stimulated without blocking Ab, was measured at 3 h. We found that Cav1-KO CD8 T cells were more readily inhibited than Cav1-WT cells by addition of anti–LFA-1 Ab at all time points up to 120 min after peptide stimulation (Fig. 6E). Moreover, titration of the blocking anti–LFA-1 Ab showed that Cav1-KO CD8 T cells were inhibited by lower concentrations of Ab than were Cav1-WT cells (Fig. 6F). Taken together, these data suggest that Cav1 plays a role in the dynamic regulation of molecules within T cell membranes, and in particular influences LFA-1 redistribution to the IS and thus the avidity of its interaction with ligand with consequent impairment of T cell activation.

## Discussion

Cav1 has been shown to influence membrane dynamics and modulate cell adhesion, cytoskeletal organization, and polarity in a variety of cell types (62, 63). In this study, we show that lack of Cav1 in primary CD8 T cells directly impacted the lipid content of the T cell membrane, with a specific overall reduction in the abundance of short-chain fatty acid sphingomyelins and cholesterol. Additionally, Cav1-KO CD8 T cells showed several changes from WT T cells upon stimulation through the TCR, including an altered morphology and an inability to appropriately redistribute key molecules, including integrins and Lck. Moreover, Cav1-KO CD8 T cells responded less efficiently to Ag stimulation and showed defects in the acquisition of effector function, as has been documented previously (32).

Our data suggest that Cav1 plays a role in influencing the distribution of molecules in the plasma membrane following TCR stimulation. Migrating T cells establish a polarized morphology, with an actin-rich leading edge and a uropod formed at the trailing edge, which is required for efficient crawling. LFA-1 distributes between both poles of the cell, participating in the pulling or pushing of the cell within the migrating gradient. Similarly, during cell migration Cav1 has been reported to accumulate at the anterior, leading edge of cultured fibroblasts (64), smooth muscle cells (65), and endothelial cells (66), as well as at the posterior, trailing edge of bovine aortic endothelial cells and fibroblasts (67, 68). Furthermore, Cav1 has been shown to influence cell polarity directly. Cav1-deficient fibroblasts displayed loss of polarized cell morphology with associated removal of focal adhesions at cell edges and loss of directional cell migration upon stimulation, defining an essential role for Cav1 in cell adhesion and motility of fibroblasts (49). We found that Cav1-KO CD8 T cells displayed reduced spreading with altered cellular morphology upon interaction with ICAM-1. Our data indicate that Cav1 is involved also in CD8 T cell polarity, as uropod formation was compromised in TCR-stimulated Cav1-KO cells migrating on ICAM-1, as was the redistribution of Ezrin and Lck. Flotillins (caveolae-associated membrane proteins) have been shown to have a role in uropod formation in neutrophils through direct interactions with the cortical cytoskeleton through myosin-IIa (69). Indeed, Filamin-A, a ligand of Cav1, has been shown to interact with β_2_ and β_3_ integrins through cytoskeletal association (70–73). Using coimmunoprecipitation followed by Western blot, we were unable to detect a direct association between Cav1 and Filamin A, Csk, Fyn, or CD18, all of which have been implicated in mediating TCR-induced signals in T cells and in regulating membrane dynamics in association with Cav1 in other cell models (74–77). However, owing to the very low abundance of Cav1 in primary T cells compared with other cell types, the association of other molecules with Cav1 may be below the level of detection by these biochemical assays.

Cav1 has been shown to impact membrane dynamics by directly binding cholesterol and sphingolipids at the plasma membrane (44, 62) or through coordination of signaling proteins that regulate the actin cytoskeleton (62, 78). Moreover, Cav1 has been shown to transport cholesterol from the endoplasmic reticulum to the plasma membrane in fibroblasts, thereby regulating the abundance of cholesterol in the plasma membrane (44). The reduction in the abundance of cholesterol and sphingolipids in Cav1-KO CD8 T cells suggested that Cav1 might play a similar role in T cells. Upon activation we observed Cav1 redistribution from the plasma membrane, in naive T cells, to an almost exclusive location within a detergent-resistant cellular pellet that is highly enriched in lipids and cytoskeletal components, consistent with a dynamic role for Cav1 in membrane homeostasis.

In CD8 T cells the abundance of cholesterol in the membrane directly influences T cell function, with increased cholesterol potentiating effector function and, conversely, depletion of cholesterol-impairing function (45). Thus, the reduced responsiveness of Cav1-KO OT-1 T cells is consistent with the reduction in cholesterol and sphingomyelin content of the cells we observed. LFA-1 has been postulated to associate specifically with a subset of lipid rafts that are high in cholesterol in primary T cells, and this association regulated clustering and LFA-1–mediated adhesion (79). Other studies also correlated LFA-1 activity and lipid raft localization by showing the clustering of high-affinity LFA-1 (by exposure to the divalent cation Mn^2+^ or following domain I removal) and high-avidity LFA-1 (by exposure to PdBu) within lipid rafts, as well as the exclusion of inactivated LFA-1 by actin cytoskeleton associations (80–82). Our data are most consistent with the absence of Cav1 influencing LFA-1 avidity, which is promoted by redistribution and thus increased valency, rather than by changes in affinity arising from repositioning or conformation of the integrin I domains to regulate LFA-1 adhesion. We found that in Cav1-KO CD8 T cells the CD11a subunit of LFA-1 as well as F-actin were less efficiently relocated to the IS, which is an area high in lipid raft content, supporting the view that Cav1 may be involved in organization of these signaling-rich regions in T cells. A lessening in LFA-1 avidity would also account for the reduction in homotypic adhesion and reduced adhesion under flow observed after TCR stimulation of Cav1-KO T cells. Collectively, this reduction in LFA-1 adhesion was insufficient to abort T cell signaling but could account for the impairment in T cell responses to Ag as shown in this study and previously (32).

The inability to detect caveolae-like structures on naive CD8 T cells and the relatively low levels of Cav1, Cav2, and PTRF transcript in lymphoid organs compared with adipose tissue, which is enriched in the specialized membrane microdomains, suggest that in T cells, Cav1 plays a distinctly different role than in other cell types where its main role is in caveolae formation. Indeed, the combination of proteomics with subdiffraction-limit microscopy has confirmed that Cav1 scaffolds are structurally and functionally distinct from caveolae (30). Expression of Cav1 at low levels can result in the assembly of stable oligomerized Cav1 microdomains, or scaffolds, comprised of ∼15 Cav1 molecules (78, 83, 84). However, the role of noncaveolar Cav1 remains elusive.

In conclusion, we report that Cav1 is involved in the transduction of TCR signals required for optimal regulation of integrin adhesion and function in primary CD8 T cells. These findings support the emerging view that membrane adaptor proteins serve as checkpoints for controlling signaling thresholds and cellular output. TCR signaling pathways are not linear but diverge, facilitated by numerous membrane adaptors, which may include Cav1. Integrin signaling is fundamental to T cell activation and plays a major role in the formation of the IS, cell adhesion, and migration. Cav1 is clearly involved in these processes in primary T cells and is therefore a potential target for manipulation of TCR signals to improve T effector function during an immune response.

## Supplementary Material

Data Supplement
