# Supplementary material for: Caveolin-1 Influences LFA-1 Redistribution upon TCR Stimulation in CD8 T Cells
Source: J Immunol. 2017 Jun 21;199(3):874–84. doi: 10.4049/jimmunol.1700431 (PMC5523581; doi:10.4049/jimmunol.1700431)
Supplement: Data Supplement [file JI_1700431.zip › JI_1700431_Supplemental_Figures_1.pdf]

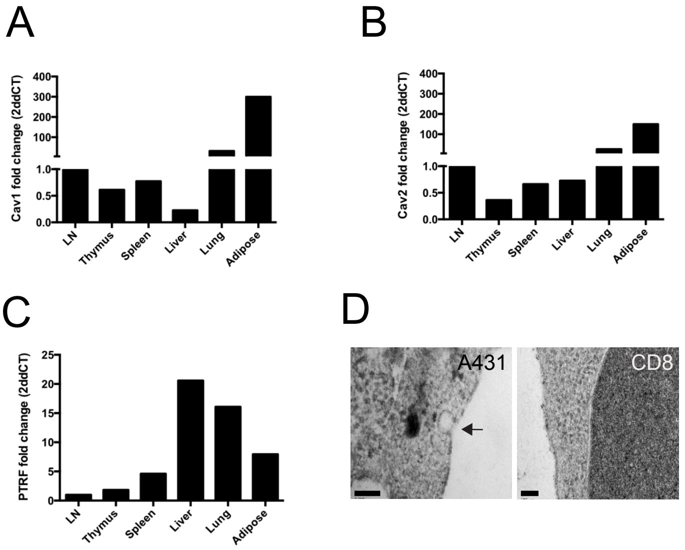

Supplementary Figure 1. Lymphoid organs express low levels of Cav1, Cav2 and PTRF mRNA. Fold change relative to LN of (A) Cav1, (B) Cav2 and (C) PTRF mRNA levels normalized to 18S RNA in secondary lymphoid and non-lymphoid organs. Data is representative of 1 or 3 independent experiments. (D) EM identification of caveolae-like structures (indicated by arrow) in A431 cells but not naïve CD8 T cells. Scale bar 100nm.

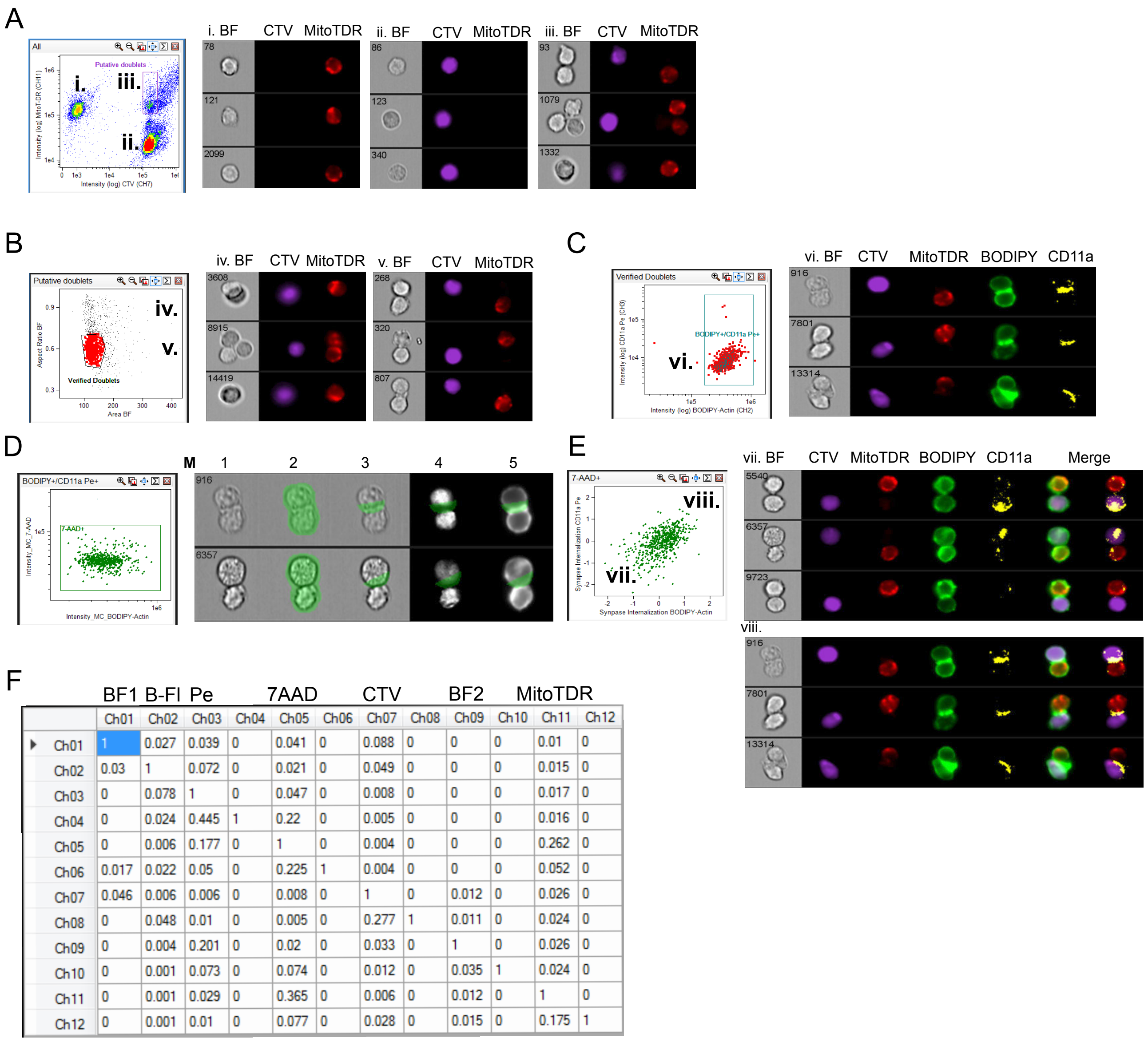

F

	BF1	B-FI	Pe		7AAD		CTV		BF2		MitoTDR	
	Ch01	Ch02	Ch03	Ch04	Ch05	Ch06	Ch07	Ch08	Ch09	Ch10	Ch11	Ch12
Ch01	1	0.027	0.039	0	0.041	0	0.088	0	0	0	0.01	0
Ch02	0.03	1	0.072	0	0.021	0	0.049	0	0	0	0.015	0
Ch03	0	0.078	1	0	0.047	0	0.008	0	0	0	0.017	0
Ch04	0	0.024	0.445	1	0.22	0	0.005	0	0	0	0.016	0
Ch05	0	0.006	0.177	0	1	0	0.004	0	0	0	0.262	0
Ch06	0.017	0.022	0.05	0	0.225	1	0.004	0	0	0	0.052	0
Ch07	0.046	0.006	0.006	0	0.008	0	1	0	0.012	0	0.026	0
Ch08	0	0.048	0.01	0	0.005	0	0.277	1	0.011	0	0.024	0
Ch09	0	0.004	0.201	0	0.02	0	0.033	0	1	0	0.026	0
Ch10	0	0.001	0.073	0	0.074	0	0.012	0	0.035	1	0.024	0
Ch11	0	0.001	0.029	0	0.365	0	0.006	0	0.012	0	1	0
Ch12	0	0.001	0.01	0	0.077	0	0.028	0	0.015	0	0.175	1

Supplementary Figure 2. Imagestream analytical workflow for identifying 1:1 heteroconjugates and measuring CD11a in the IS. (A) Bi-varate plot of integrated CTV fluorescence intensity (x) versus same for MitoTDR intensity (y). Gate identifies putative double stained events. Example multi-spectral images shown below for each major region (i-iii). (B) Bi-varate plot of BF Area (x) versus Aspect Ratio of the same (y). Gate captures events formed from two conjoined cells. Example multispectral images are shown from outside this gate (iv) and within (v). (C) Bi-varate plot of integrated BODIPY-Fl fluorescence intensity (x) versus the same for CD11a Pe (y). Gate captures all double positive events. Example multispectral images are shown from within this gate (vi). (D) Cells also gated for 7-AAD positivity prior to constructing the interface mask shown in panel M. Mask key: 1. BF image (CH01) no mask shown; 2. BF image (CH01), default mask (M01) overlaid; 3. BF image (CH01), synapse mask, Interface (M07, M05, M08) mask overlaid; 4. 7-AAD image (CH05), Interface (M07, M05, M08) mask overlaid; 5. BODIPY-Fl image (CH02), Interface (M07, M05, M08) mask overlaid. (E) Internalization score for each verified conjugate was calculated for BODIPY-Fl (vii) and CD11a (viii). Median internalization score was reported. (F) A typical matrix spillover coefficients is presented, calculated from the IDEAS compensation wizard using single stained controls (BODIPY-Fl (B-Fl), Pe, 7-AAD, CTV and MitoTDR).
